# Bilateral Spontaneous Renal Hematoma or Wünderlich Syndrome: A Rare and Life-Threatening Clinical Entity

**DOI:** 10.7759/cureus.89848

**Published:** 2025-08-11

**Authors:** Luis Fernando Muñoz Chavez, Luis Montiel López, Ivan Alejandro Elizalde Uribe, Leonor Bonilla Quezada, Betsy Margot Lopez Gomez

**Affiliations:** 1 Internal Medicine, Centro Médico Nacional 20 de Noviembre, Mexico City, MEX

**Keywords:** abdominal pain, bilateral, case report, idiopathic, renal hematoma, spontaneous

## Abstract

Wünderlich syndrome (WS) is an unusual clinical condition defined by spontaneous perirenal space hemorrhage or subcapsular renal hemorrhage without antecedent of traumatic history, often triggered by various factors. It manifests mainly as abdominal pain and fever. As the signs and symptoms are nonspecific, imaging studies are essential for diagnosing and ruling out differential diagnoses. We present a case of a 42-year-old female with no previous pathological or traumatic history, who was admitted with abdominal pain and episodes of fever, attributed to bilateral spontaneous renal hematomas. We conducted a comprehensive clinical history to rule out metabolic, autoimmune, infectious, traumatic, or congenital factors. Laboratory tests for autoimmune, infectious, coagulation, and metabolic causes were all negative. An abdominal contrast-enhanced computed tomography revealed a bilateral renal hemorrhage, ruling out other abdominal causes of pain and confirming a bilateral WS. The patient underwent fluoroscopy-guided drainage and biopsy because we did not have an accurate diagnosis based on the imaging reports, which indicated either pure hematomas or renal abscesses. We ruled out any tumoral or infectious process with the drainage samples. After a thorough consideration and elimination of all possible causes, we arrived at the diagnosis of an idiopathic bilateral WS, an infrequent medical occurrence.

## Introduction

Wünderlich syndrome (WS), an exceptionally rare and unique clinical emergency condition, is characterized by spontaneous perinephric space and subcapsular renal hemorrhage in patients without a traumatic event [1,2]. 

Wunderlich first described this pathology in 1856 and has since been associated with several etiologies, such as metabolic, infectious, traumatic, vascular-autoinmune, or congenital factors. Benign or malignant neoplasms are the most common causes, with angiomyolipoma being the most common renal neoplasm responsible for the syndrome; Renal cell carcinoma is the most common malignant neoplasm that can cause spontaneous perinephric hemorrhage. Although rare, other renal tumors such as fibroma, metastases, oncocytoma, renal sarcomas, Wilms tumor, and transitional cell carcinoma can also manifest spontaneous perinephric hemorrhage [1]. Vascular disease was the second most common cause, with polyarteritis nodosa occurring most frequently. Infections such as pyelonephritis, abscess, or infection-associated inflammatory erosion of the renal vessels with intravascular thrombosis and renal parenchymal necrosis have also been identified as common causes [1]. Moreover, cystic renal diseases could be a potential cause; sometimes, simultaneous rupture of the cysts may occur in the pelvicalyceal system and perinephric space [1]. Rare etiologies include end-stage renal disease, the use of systemic anticoagulation, nephrosclerosis, and idiopathic WS, which is a case without any underlying kidney abnormality, occurring in approximately 5% to 10% of patients [1,3].

Clinical presentation includes abdominal pain, which is present in 67% of patients, hematuria (40%), a flank mass, and hypovolemic shock (27%). The characteristic Lenk's triad of palpable flank mass, abdominal pain, and hypovolemic shock is observed in only 20% of patients with the syndrome [2, 4]. The presence of this triad is highly suggestive of WS and should prompt immediate investigation and management.

Imaging studies, such as computed tomography (CT), magnetic resonance imaging (MRI), and ultrasound (US), are crucial in the approach to patients presenting with symptoms of WS [2]. The Shah JN, et al. article [2] mentions that the CT of the abdomen and pelvis is often the initial modality of choice and is pivotal to diagnosis and management of WS, it helps in the detection of hemorrhage, allows accurate estimation of its extent, and assists in the differentiation of a hematoma from an underlying mass, and the identification of many of the underlying causes. MRI is commonly performed to detect causes of WS in patients in whom the initial or follow-up CT examination did not allow identification of the bleeding source [2]. Although CT is the standard imaging technique for evaluation of patients with WS, US is highly sensitive for the identification of a perirenal hematoma. It is helpful in follow-up examinations, where it can assess the evolution of the hematoma and provide real-time guidance for percutaneous drainage of the hematoma, thereby playing a crucial role in monitoring the progression of the disease [2].

We present this case report of a woman patient with no previous pathological or traumatic history, based on our comprehensive approach in the clinical aspect. Abdominal pain was the primary symptom guiding the diagnostic approach, followed by laboratory and imaging studies to rule out other differential diagnoses and identify patterns consistent with probable etiologies. Performing this comprehensive diagnostic approach in the face of this rare entity with an atypical presentation helped us implement the appropriate treatment for our patient, serving as a reference for future cases presenting with similar findings.

## Case presentation

A 42-year-old female patient, previously healthy and without a traumatic history, arrived at the emergency service of the medical center with a one-week sudden, severe, dull pain in the epigastrium, which radiated to the right and left iliac fossa, subsided with the use of analgesics and rest. The pain was accompanied by nausea, vomiting, headache, and self-limiting fever without the need for any mitigating factors such as antipyretic medications and physical cooling methods. 

Vital signs upon admission were as follows: blood pressure 128/68 mmHg, temperature 36.7 °C, oxygen saturation 95%, heart rate 61 beats per minute, respiratory rate 20 breaths per minute, height 168 cm, weight 110 kg. On physical examination, notable findings included a painful abdomen on superficial and deep palpation in both iliac fossae; the right Giordano sign was positive, and telangiectasias were in both lower limbs.

Admission laboratory results, reference values, and patient basal values are in Table 1. A drop in hemoglobin and an increase in serum creatinine, with normal serum electrolytes, were identified.

**Table 1:** Laboratory studies.

Test	Admission values	Post-treatment value	Range values
Leukocytes	12.71	8.04	5-10 thousand/mm^3^
Neutrophils	10.39	5.57	1.4-6.5 thousand/mm^3^
Lymphocytes	1.35	1.85	0.1-3.4 thousand/mm^3^
Hemoglobin	10.3	12.7	12-16 g/dL
Hematocrit	34.4	38.3	37%-47%
MCV	80	77.8	82-96 fL
MCH	24.7	25.8	27-31 pg
Platelets	264	327	150-450 thousand/mm^3^
Glucose	103	86	74-106 mg/dL
Creatinine	1.83	0.73	0.1-1.3 mg/dL
Urea	41	19.2	19-49 mg/dL
Sodium	137	136	132-146 mEq/L
Potassium	3.63	4.0	3.5-5.5 mEq/L
Chloride	104.9	108	99-109 mEq/L
Calcium	8.4	9.40	8.3-10.6 mg/dL
Phosphorus	4.1	3.30	2.4-5.1 mg/dL
Magnesium	2.06	2.02	1.3-2.7 mg/dL
Prothrombin time	13.4	12.1	11-15 seconds
INR	1.15	0.9	0.8-1.1
Partial thromboplastin time	25.4	26	25-33 seconds
Thrombin time	15	16.5	15-20 seconds
Lactate dehydrogenase	131	135	120-246 UI/L
Procalcitonin	<0.5	<0.5	<0.5 low risk; >2 high risk
Erythrocyte sedimentation rate	60	9	0-10
C-reactive protein	89.2	1.1	0-3 mg/L

**Figure 1:**
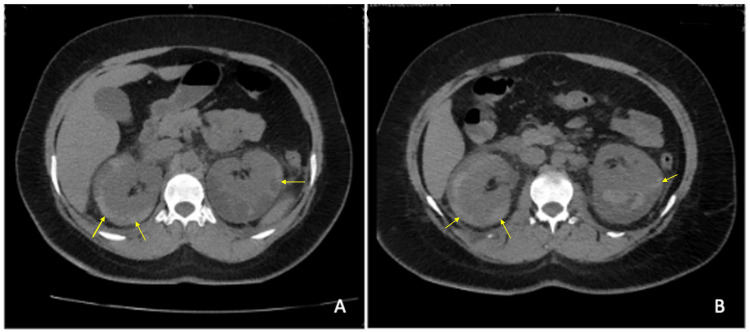
Simple abdominal computed tomography. Liver of 20.5 cm homogeneous density without lesions; right kidney 12.7 cm heterogeneous density, with inflammation of Gerota's fascia; left kidney 11.2 cm heterogeneous density with evidence of inflammation of Gerota's fascia; inflammation of the bilateral perinephric space; thickening of the transverse and descending colon, probable image of diverticula; (A) axial section, upper third of the kidney; (B) axial section of the lower third of the kidney. Yellow arrows indicate inflammation of Gerota's fascia.

As we encountered a clinical presentation with no significant history that did not indicate a specific pathology, the emergency medical team, together with our internal medicine team, agreed to perform a CT scan as the first choice study, especially to rule out any pathology requiring urgent surgical intervention. The CT revealed bilateral renal enlargement with heterogeneous parenchyma, a perirenal space with a mixed image of echogenic and anechoic content, and no vascularity. The left kidney showed signs of inflammation of Gerota's fascia and both perinephric spaces (Figure 1). 

**Figure 2:**
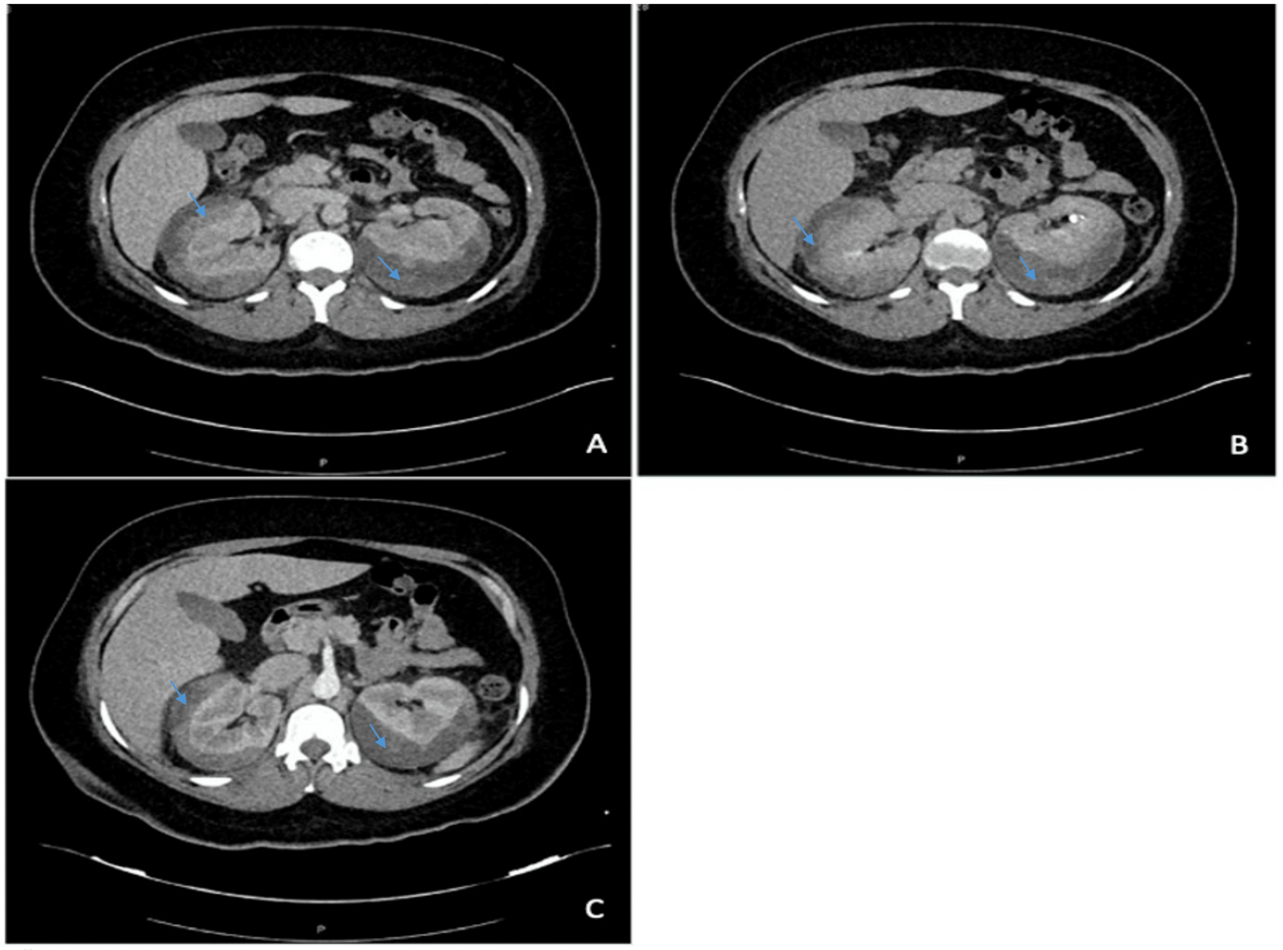
Contrasted kidney tomography. Axial section, both kidneys increased in size with hyperdense areas. Blue arrows indicate heterogeneous image with a range of 40-60 HU suggestive of bilateral renal hematomas or bilateral renal abscesses. (A) Arterial phase; (B) parenchymatous phase; (C) late phase.

**Figure 3:**
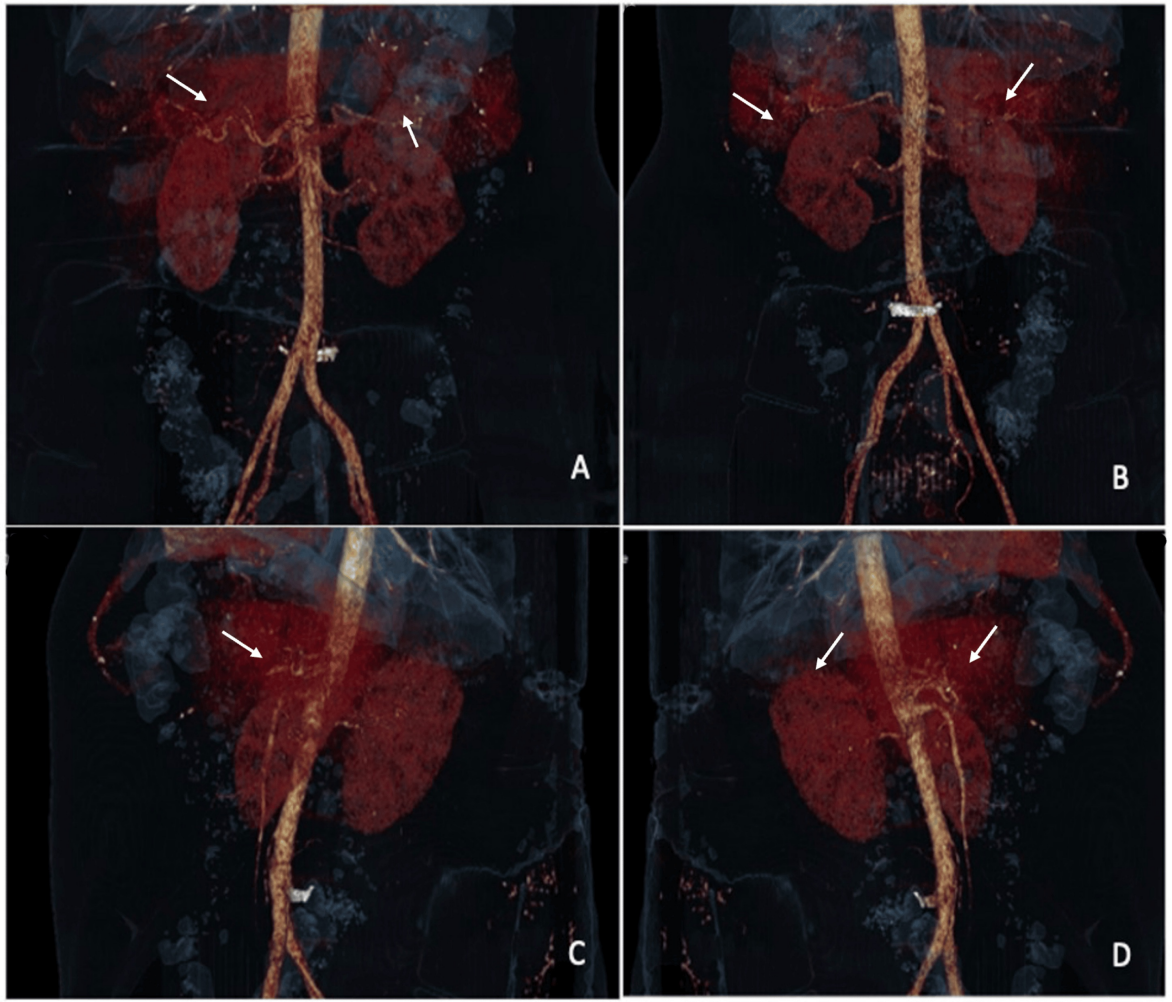
Contrasted abdominal tomography reconstruction. Both kidneys increased in size with hyperdense areas. White arrows indicate heterogeneous areas suggestive of hematomas or abscesses. (A) Anterior view; (B) posterior view; (C) right view; (D) left view.

CT of the kidneys with contrast revealed a heterogeneous image with a range of 40-60 Hounsfield units (HU) that surrounded and displaced both renal units, suggestive of bilateral renal hematomas or kidney abscesses (Figure 2). Abdominal CT reconstruction was performed, showing heterogeneous images at the renal level, with the appearance of collections suggestive of hematomas but not ruling out the possibility of the presence of renal abscesses (Figure 3).

During her clinical course, she presented with a decrease in her hemoglobin level of up to 2 g in one day. Despite this, she maintained hemodynamic stability. The official imaging reports were not conclusive, as the HUs of the collections could indicate a case of renal hematomas. However, they were also compatible with the possibility of abscesses. Recognizing the limitations of imaging studies alone, we proceeded with a renal biopsy and CT-guided drainage of bilateral hematomas to obtain samples for cytochemical, pathological, and culture studies, which will allow us to determine whether we were dealing with bilateral hematomas or renal abscesses. The procedure yielded 130 cc from the left side and 80 cc from the right side, without complications. After the procedure, the patient remained asymptomatic.

The cytochemical examination was compatible with pure hematological content with mild non-specific inflammatory cells. In addition, Gram stains did not detect microorganisms. The bacterial and fungal cultures of both sides did not have microbiological development. GeneXpert for *Mycobacterium tuberculosis* was also performed, with a negative report. With these results, we conclude that the renal collections were hematomas. The renal biopsy of both sides were negative for benign or malignant neoplasms. Autoimmune disease was ruled out when the laboratory reported negative serologies for vasculitis, antiphospholipid syndrome and systemic lupus erythematosus. The approach to polyarteritis nodosa was carried out, with muscular and skin biopsy being negative, tests for Hepatitis B and C being negative, and a serological profile for vasculitis being negative, ruling out the diagnosis. Additionally, we performed a renal angiography, which revealed no vascular alterations. We got normal coagulation, Von Willebrand factor, and thyroid function tests. We investigated all possible causes, and despite addressing each one, we concluded that it was an idiopathic non-typical presentation of WS.

One week before the drainage, the leukocytes returned to normal values, the C-reactive protein decreased, and kidney function improved. The patient also experienced no further abdominal pain or fever. The patient was discharged home due to clinical improvement.

## Discussion

Several diseases can generate spontaneous renal hematomas, the most commonly associated etiologies are benign tumors (31.5%), where angiomyolipoma is the primary cause, malignant tumors (29.7%) with renal cell carcinoma being the most frequent, and vascular diseases (12.1%), with polyarteritis nodosa being the most common cause, due to rupture of aneurysms caused by the deposit of immune complexes in the walls of small and medium-caliber arteries [3,4]. Other less frequent causes include pyelonephritis, hydronephrosis, coagulation disorders, prolonged treatment with corticosteroids, pheochromocytoma, and acute or chronic rejection of transplanted kidneys. It is frequently related to hypertension (33%-50%) and atherosclerosis (80%-87%) [4,5,6]. While in most cases the etiology is identified, in a significant number of patients, it remains unknown and classified as idiopathic. The term *idiopathic* in this context means that the cause of the renal hematoma is unknown or cannot be determined using current diagnostic tools and knowledge. As reported in the meta-analysis by Zhang et al. [3], which included 47 studies and 165 cases, 11 studies (6.7%) had an undetermined etiology [3]. We listed the various etiologies in Table 2. 

**Table 2:** Etiologies of Wünderlich syndrome.

Neoplasms	Vascular-autoimmune conditions	Cystic renal diseases	Infections	Rare conditions
Angiomyolipoma, renal cell carcinoma, fibroma, sarcomas, metastases, and transitional cell carcinoma	Polyarteritis nodosa, renal artery aneurysms and pseudoaneurysms, arteriovenous malformations and fistulas, and renal vein thrombosis	Simple and hemorrhagic cysts, acquired cystic kidney disease, and autosomal dominant polycystic kidney disease	Pyelonephritis, abscess, and emphysematous pyelonephritis	Idiopathic syndrome, nephrosclerosis, systemic anticoagulation, and end-stage renal disease

Contrary to the common belief, the classical clinical triad, which includes unilateral flank pain, hypovolemic shock, and a palpable lumbar mass (Lenk's triad), is a rare occurrence. Kim et al. [4] reported fever and hematuria in most cases, with only 20% of patients presenting the complete clinical triad [3,4].

In the diagnostic process, imaging studies play a crucial role. CT is the gold standard due to its ability to provide precise details, such as dimensions, shape, and Hounsfield units, of the affected area in spontaneous renal hematomas [2]. A review by Katabathina et al. [1] suggests that, in suspected cases of WS, the goals of imaging include confirming the diagnosis by identifying renal hemorrhage in the subcapsular and/or perirenal space, as well as comprehensively assessing the possible cause of the bleeding [1, 2]. The abdominal CT scan is a cost-effective and readily available study that allows for a detailed analysis of the kidneys, which enables the characterization of WS [1,6]. The CT sensitivity is close to 100% in demonstrating perirenal hemorrhage [6,7]. It is also more accurate than US in evaluating the severity of the hemorrhage and its underlying cause. However, Badiola et al. [7] reported that the sensitivity of CT in detecting the etiology of WS at the time of the acute hemorrhage has been reported to be around 57%. In cases in which the lesion causing the perirenal hemorrhage has not been confirmed, RM or follow-up CT is recommended [7]. The study by Shah et al. [2] underscores the importance of multiphasic CT and MRI in diagnosis. These imaging techniques are the mainstays of diagnosis, as they enable the detection of the cause of spontaneous hemorrhage and provide a roadmap for interventional radiology or emergency surgery. Even in cases where the initial CT or MRI fails to establish the cause of hemorrhage, multiphasic follow-up CT or MRI after the acute phase of WS resolves can allow for an accurate diagnosis. This precision in diagnosis is crucial, as it guides the choice of intervention and is a key factor in the optimal treatment of patients with life-threatening renal and perirenal hemorrhage [2,8].

The first step of the treatment is hemodynamic stabilization with volume resuscitation using intravenous fluids, including solutions or blood products, and immediate reversal of anticoagulation [5]. Conservative management with angiotensin receptor blockers to prevent complications of secondary hypertension, and the use of antibiotics if an infectious etiology is strongly suspected, are recommended [2]. Dongming et al. [8] mention that percutaneous drainage of abscesses or hematomas is recommended in cases where an infectious etiology, such as an abscess, is suspected; it offers a means to alleviate symptoms and decrease renal pressure. Should this approach fail to provide sufﬁcient drainage, surgical intervention for incision and drainage becomes necessary to remove the accumulation of ﬂuid, pus, or blood [8]. Surgery is also reserved for hemodynamically unstable patients and those with neoplastic disease [9,10]. Treatment such as renal artery embolization or nephrectomy is indicated depending on the severity [11].

The anatomopathological study is essential for determining the etiology of renal hemorrhage, as the most common etiologies are renal neoplasms and kidney vascular diseases [3,11].

## Conclusions

WS broadens the differential diagnosis for abdominal pain, underscoring the importance of awareness and thorough diagnostic evaluation. Its nonspecific presentation necessitates careful exclusion of other conditions through imaging and complementary studies, which are the key to identifying differential diagnosis and underlying etiologies, including the rare ones such as idiopathic cases. Treatment strategies for WS depend on the patient's hemodynamic stability and the presence of complications or possible etiologies involved. From our perspective, recognizing this syndrome's variable clinical manifestations and potential causes is vital for timely, accurate diagnosis and management, ultimately improving patient outcomes.
